# What role can videogames play in the COVID-19 pandemic?

**DOI:** 10.35241/emeraldopenres.13727.2

**Published:** 2020-10-05

**Authors:** Hannah R. Marston, Rachel Kowert

**Affiliations:** 1 Health and Wellbeing Strategic Research Area, Open University, UK, Milton Keynes, Buckinghamshire, MK7 6AA, UK; 2 Take This, Seattle, WA, USA

**Keywords:** Digital games, Social connectedness, Mental health, PTSD, Interaction, Intergenerational, Childless, Ageing

## Abstract

Video games are often thought of as trite activities for younger generations. However, research in game studies over the last few decades have revealed that games can be valuable tools for growth and connection, particularly among older generations. Exploring the ways digital games can be used as tools for connection has gained increased attention in recent months with global quarantines as a result of COVID-19. This article reviews the research that has examined the utility of digital games for older adults, focusing specifically on the ways in which games can be tools for social connectedness and psychological healing for older adults and intergenerationally. Special focus will be placed on the role games can play for post-traumatic stress among first responders.

## Introduction

Videogames have become a phenomenal form of entertainment over the last 60 years and their history has been documented through various texts ( Forster, 2005; Herman, 2001; Kent, 2000). Scholarly activity and research have illustrated this growing interest from academe, with many scholars focusing on the impact, user experience and design of videogame, as illustrated by Marston & del Carmen (2020) in their recent scoping review that focuses on the Generation X cohort. This cohort has to date received little attention from academe ( Brown & Marston, 2018), unlike the Baby Boomer cohort, which has received substantial interest from scholars across the fields of gerontology, gerontechnology, media and communications.

One sub-domain of the Game Studies discipline has garnered interest: the Games for Health (G4H) movement. G4H actively facilitates interdisciplinary research in a bid to achieve the primary aims and objectives of this domain, and to date scholarly activity has shown how videogames can be designed, developed and used for a myriad of health conditions. For example, obesity ( Lu *et al.*, 2013), fall prevention ( Marston *et al.*, 2015), dementia ( Cutler *et al.*, 2016), and social connectedness ( Schell *et al.*, 2016). Since 2010, there has been several scholarly reviews published, with the focus on the benefits of videogames over the last decade ( Bleakley *et al.*, 2015; Hall *et al.*, 2012; Marston *et al.*, 2016; Marston & Smith, 2012; Miller *et al.*, 2014).

The purpose of this opinion piece is to discuss the contemporary landscape of videogames and the relationship that they can and do play from the standpoint of COVID-19.

## Intergenerational, social connectedness and loneliness

From the standpoint of intergenerational gaming ( Marston & Azadvar, 2020), over the last decade there has been a growing interest in videogames from this perspective. Contemporary literature illustrates this growth, with a systematic review published in 2017 ( De la Hera *et al.*, 2017), which comprised of 16 papers. This review highlighted four categories: (1) reinforcing family bond, (2) enhancing reciprocal learning, (3) increasing understanding of the other generation, and finally (4) reducing social anxiety. In addition, research conducted by Voida & Greenberg (2010); Voida & Greenberg (2009) purport positive design challenges by fostering intergenerational gaming practices. Similarly, De la Hera & colleagues’ (2017) research aligns with the notions of Voida & Greenberg (2012), and also reason the positive relations between intergenerational gaming and design by understanding the barrier and enablers to existing game console design.

There has been a swathe of contemporary research associated with social connectedness and loneliness by scholars in the field of Gerontology, positing various social factors relating to the experience of social and emotional loneliness, and a disconnect with members of the community and society ( Drennan *et al.*, 2008; Heylen, 2010).

de Jong Gierveld (1998) defines loneliness as an unpleasant and negative feeling, especially when there is a perception of disconnection between achieving and desiring the quality and/or quantity of social connections. Additionally, Wenger & Burholt (2004) have noted that social isolation is an objective measure associated to the dearth of social contact/connections. Markers and experiences in our lives, such as retirement, bereavement, illness, disability or caring responsibilities, can be triggers to loneliness. With this in mind, the COVID-19 pandemic has exacerbated loneliness and social isolation amongst society, not only amongst older populations ( Drennan *et al.*, 2008; Ejlskov *et al.*, 2020; Marston & Morgan, 2020; Yang & Victor, 2011), but also younger cohorts ( Ejlskov *et al.*, 2020), young disabled adults ( Morris, 2001), men ( Ratcliffe *et al.* (2019) and childless adults who in/voluntary experience childlessness ( Hadley, 2020; Hadley, 2018a; Hadley, 2018b; Hadley, 2019). Hadley (2018b) argues,

“While many age related issues such as isolation, loneliness and dementia have recently gathered extensive attention (and funding) people ageing without children is a subject that remains unreported, under-researched and under-represented at all levels” ( Hadley, 2018b, p. 76–77).

Furthermore, a tri-country study ( Kendig *et al.*, 2007; Kendig *et al.*, 2010) ascertained associations between childless men and poor health, such as depression, excessive smoking, drinking and difficulty sleeping. Dykstra & Hagestad (2007) state,

“The childless ‘are vulnerable - a group at risk of social isolation, loneliness, depression, ill health and increased mortality’” ( Dykstra & Hagestad, 2007, p. 1288).

In recent weeks, contemporary research posits the potential benefits of technology for all citizens during the COVID-19 pandemic ( Marston *et al.*, 2020; Sheerman *et al.*, 2020; White *et al.*, 2020). In addition to existing narratives and discourse, technology is playing a pivotal role in various ecosystems as a means of continuing and enhancing social connections, be it amongst young gamers and/or from an intergenerational standpoint ( De la Hera *et al.*, 2017; Marston & Azadvar, 2020; Voida & Greenberg, 2010; Voida & Greenberg, 2009; Wang *et al.*, 2018).

Conversely, does technology and videogames play a greater significant role in the lives of childless middle-and-older adults? To date, there is an absence in the Game Studies, Gerontology and Gerontechnology literature surrounding the benefits and barriers to using technology by adults who are childless.

## Health and wellbeing for emergency responders and frontline workers

There has been growing scholarly activity surrounding the health, wellbeing and post-traumatic stress (PTSD) of emergency services personnel (ESP) in the UK. Contemporary research suggests PTSD is greater in ESPs than in the general population ( Arble & Arnetz, 2017; Brooks *et al.*, 2019; Counson *et al.*, 2019; Mildenhall, 2019; Varker *et al.*, 2018).

Currently, research is starting to illustrate and clarify how ESPs are becoming more disproportionately exposed to specific experiences/situations in conjunction to a more overall general working environment. In the context of COVID-19, Mildenhall (2020) offers guidance in the area of psychosocial and mental wellbeing, primarily aimed at paramedics, personnel and managers, while from a policing perspective, Hesketh *et al.* (2018) offers guidance associated to PTSD, targeting police personnel across the UK.

In a forthcoming scoping review, Marston *et al.* (2020) demonstrate the paucity in contemporary literature surrounding the design, use and deployment of specific technologies, such as mobile health (mHealth) apps, targeting ESPs, specifically police personnel and support staff. Conversely, the Blue Light Wellbeing Framework ( Hesketh & Williams, 2017) has been designed with the objectives of health and wellbeing provision for both uniformed and support staff. A web portal – Oscar Kilo (OK), deployed for the National Police Wellbeing Service (NPWS) – comprises of a set of independent standards, aimed at police personnel and ESPs, and affords organisations and police forces the opportunity to audit and benchmark themselves against this framework.

The OK framework was co-designed by a myriad of actors (e.g. practitioners (all levels), professionals and academics), and includes five areas: (1) strategic and tactical planning templates, (2) psychological risk management guidance ( Hesketh *et al.*, 2017), (3) responding to trauma guidance ( Hesketh & Tehrani, 2019), (4) the GAIN pyramid ( Hesketh *et al.*, 2017), and (5) a series of real stories presented in animations. Whilst there is a growing body of evidence, there is still little scholarly evidence to understanding how technologies, such as mHealth apps and/or videogames, can facilitate health, wellbeing and PTSD to front line ESPs and support staff.

The use of videogames to facilitate support and offer treatment for PTSD ( Holloway & Reger, 2013; Macleod & Sloan, 2017) has garnered greater attention in recent years, with the use of videogames and online virtual environments. While the online virtual environment – Second Life, 2020 ( https://secondlife.com/) has previously been used as a tool to offer support to military personnel and their families in an attempt to alleviate PTSD ( Hemmerly-Brown, 2019). From a US military personnel perspective, a study by Colder Carras *et al.* (2018) has been performed, comprising of 20 participants who engaged with videogames as a means of understanding mood and stress levels. Overall, this qualitative study reported positive results by veterans associated with behavioural recovery and PTSD health. The respective authors suggested videogames can act as a form of personal medication, as a way of promoting recovery ( Colder *et al.*, 2018, pg. 2). While a systematic review ( Callejas-Cuervo *et al.*, 2017) conducted in 2017 and comprising of 15 articles also ascertained positive directions to rehabilitation for PTSD treatment, relating to emotional recognition and videogames.

The videogame *Tetris* has been used in the environment of an emergency department as an approach of reducing trauma after a traffic accident. Using a randomized control trial (RCT) design, Iyadurai *et al.* (2018) conducted an intervention that occurred within six hours of the incident. The control group were required to write an activity log for 20 minutes, and the intervention group were required to play Tetris for 20 minutes. Over a period of one week, the aim of the RCT was to compare the number of intrusive trauma memories. Although findings from this proof-of-concept study showed positive benefits at one week, the respective authors suggest a larger and longer trial is needed to understand the benefits at one month.

In the field of game studies scholars have explored game playing experiences ( Azadvar & Dalqvist, 2020; Bianchi-Berthouze *et al.*, 2007; Deterding, 2011; Deterding *et al.*, 2013; De Schutter, 2010; De Schutter & Brown, 2015; Ermi & Mäyrä, 2005; Marston, 2013a; Marston *et al.*, 2016; Nacke & Lindley, 2008; Nacke & Lindley, 2009; Payne *et al.*, 2011; Voida & Greenberg, 2012; Whitelock *et al.*, 2014) to understand how players engage with videogames. Generally speaking game playing experiences vary based on the respective game genre(s) been played ( De Schutter & Brown, 2015; Marston, 2013a; Marston *et al.*, 2016; Nacke & Lindley, 2008; Nacke & Lindley, 2009; Voida & Greenberg, 2012; Whitelock *et al.*, 2014). For example, players may experience deep meaningful game experiences through role-playing games, whereas simulation or puzzle games may not elicit the same experience ( Connell & Dunlap, 2020). It is also important to understand the role of player motivations on game experience. If a player is highly motivated to achieve, then Incorporating gamification elements that provide feedback on progress, such as goals rewards and leader boards, could influence their gaming experience exponentially ( Deterding, 2011; Deterding *et al.*, 2011; Deterding *et al.*, 2011; Havukainen *et al.*, 2020; Marston & Hall, 2015). Understanding the purpose or motivations of a gamer can afford the game design to be tailored specifically, especially if you are planning to design a game aimed at a specific audience ( De Schutter, 2010; Marston, 2013b; Marston *et al.*, 2015; Pearce, 2008) or rationale. Therefore, understanding the motivations of the target audience is important for game design ( Apperley, 2006; De Schutter, 2010; Elliott *et al.*, 2012; Goddard & Muscat, 2017; Havukainen *et al.*, 2020; Marston, 2013b) and potential positive outcomes via gameplay.

## Gaming in a time of COVID-19

Videogames have become a feature in the homes of many citizens old and young over the last several decades, transforming the home to one which now accepts the videogame console as a feature. Flynn (2003) describes the domestication of the home and states,

“The home is once again framed as ‘a machine for living in’ with the user most ‘at home’ when playing the game console” ( Flynn, 2003, pg. 558).

The history of videogames is long documented ( Forster 2005; Herman, 2001; Kent, 2000), detailing the move of videogames from public space into private – the home. Flynn (2003) purports how the videogame consoles over the decades has been implemented into the living space – of the digital hearth to feature and sit alongside the additional pieces of furniture in this physical space. At the time of writing this specific piece (21 ^st^ Century), Flynn pontificates and narrates the perceptions and portrayals of digital consumption 100 years earlier, describing the difference between a country house environment and prospective future housing environmental design. Whereby over the decades, one’s lifestyle, gender and media influences has led to redefining how key pieces of technology are represented and identified specifically by women ( Massey, 2000). For example,

“From this examination of the contemporary ideal home, it would appear that for the middle-class female readership of lifestyle magazines, the video console is still an alien machine in relation to narratives of identity associated with domesticity and family togetherness.” ( Flynn, 2003, pg. 565).

The notion of videogames portrayed in magazines illustrated the alternative physical space or third place (e.g. the bedroom) to game playing, rather than the living room or the digital hearth as previously described by Flynn (2003). Whereas, the previous notion of the living room as the familial space – or the suburban living room – as the gaming/meeting place was been replaced by such advertisements purported by games industry companies ( McGuire, 2003).

Existing research ( De la Hera *et al.*, 2017; Voida & Greenberg, 2010; Voida & Greenberg, 2009; Wang *et al.*, 2018) and the work presented by Flynn (2003), illustrates how videogames can enhance interaction within the physical space(s) with other gamers, and adults alike. During this unprecedented time in society, COVID-19 is impacting many physical spaces and ecosystems ( Marston *et al.*, 2020; Sheerman *et al.*, 2020; White *et al.*, 2020), be it a community group, a family, middle-aged or older adults living on their own, or a keyworker. Yet, videogames and their peripheral technologies can and do have a role to play in continuing and enhancing social connections, relationships and engagements, from within the ecosystem and/or across WiFi communications.

It is important to note the unique contribution that games provide to mediated socialization. The fact that games are playful, fun, interactive spaces differentiates them from other forms of mediated communication, such as text messaging or social media ( Kowert, 2015). They allow individuals to connect through play, which is an important facet of psychological well-being throughout the lifespan ( Connell & Dunlap, 2020). Play in and of itself is associated with reduced stress and depression, as well as a releasing of endorphins ( Robinson *et al.*, 2019). Combined with the various benefits of in-game socialization (i.e., reduced stress, depression, and sense of loneliness; see Kowert, 2015) makes games a useful tool for mitigating some of the negative impacts of COVID-19 for adults.

Taking a different route in this discussion and turning our attention to the growth of evidence positively supporting and facilitating health, wellbeing and PTSD in different populations, in addition to enhancing intergenerational relationships, further considerations are needed. For example, in this opinion piece, we have explored contemporary literature surrounding intergenerational gaming. However, for those citizens who are ageing without children or grandchildren, how can videogames facilitate positive health and wellbeing, social connections and reduce a sense of loneliness? We suggest here that future research should explore how videogames are used as a means of understanding social connections and reducing loneliness by middle-age and older adults. As previously noted by Hadley (2018b), scholarly research is underrepresented in the field of people ageing without children. Given the situation(s) that this pandemic has placed on citizens, for those who perceive themselves as gamers, and who may not have children or grandchildren, they may have already chosen to use videogames as a means of socially connecting with friends in an online environment. Using videogames as a means of socially connecting with un/known gamers may afford a person a sense of positive feeling.

From the standpoint of ESPs, evidence is growing that illustrates the need for identifying appropriate solutions for treatment of PTSD and for continuing positive health and wellbeing practices within the workforce. As noted in their forthcoming review, Marston *et al.* (2020) purport the paucity of existing literature surrounding technology use and deployment for ESPs in association with health, wellbeing and PTSD. Furthermore, they provide a series of recommendations in an attempt to move this interdisciplinary work forward, and given the current situation within society (associated to COVID-19), there is the likelihood that health practitioners, and social care keyworkers may require health, wellbeing and PTSD rehabilitation/recovering in the future. For instance, keyworkers such as those who are working in our hospitals are witnessing multiple deaths throughout their shifts, and days, and in some instance of their colleagues. Furthermore, deaths of citizens are also been reported from care/nursing homes, and many health and social care workers have chosen to ‘live in’ and shield the residents from COVID-19.

## Conclusions

In this opinion piece, we have demonstrated how videogames can play a pivotal role in various societal ecosystems from the individual/digital hearth to the larger ecosystem surrounding ESPs, health practitioners, and social care workers, who at present are exposed to various situations and trauma.

Moving this debate forward, interdisciplinary research is needed to focus on two landscapes. Firstly, from the standpoint of social sciences and how videogames can impact the lives of middle-and-older adults who are ageing without children. As previously noted, there is a paucity of research specifically focusing on adults who are ageing without children. Social connectedness and loneliness are key experiences, and more information and understanding are required to offer solutions to reduce these risks.

Secondly, more research is required from the standpoint of videogames and ESPs and the role in which videogames can be a means of reducing trauma, offering positive health, wellbeing and PTSD solutions. This would require efforts from a myriad of actors, forming a co-designed and co-produced approach to ensure all key information and take-up is included.

## Data availability

No data is associated with this article.
